# Crystal structure of 1,3-dimethyl-3-phenyl­pyrrolidine-2,5-dione: a clinically used anti­convulsant

**DOI:** 10.1107/S1600536814016717

**Published:** 2014-08-01

**Authors:** Carlos Ordonez, Ilia M. Pavlovetc, Victor N. Khrustalev

**Affiliations:** a Department of Chemistry & Biology, New Mexico Highlands University, 803 University Avenue, Las Vegas, NM 87701, USA; b Department of Engineering Photonics, St Petersburg National Research University of Information Technologies, Mechanics and Optics (ITMO University), 49 Kronverkskiy Avenue, St Petersburg 197101, Russian Federation; c X-Ray Structural Centre, A.N. Nesmeyanov Institute of Organoelement Compounds, Russian Academy of Sciences, 28 Vavilov Street, B-334, Moscow 119991, Russian Federation

**Keywords:** crystal structure, methsuximide, anti­convulsant, α-substituted cyclic imide, succinimide

## Abstract

In the title compound, C_12_H_13_NO_2_, the five-membered ring has an envelope conformation; the disubstituted C atom lies out of the mean plane through the four other ring atoms (r.m.s. deviation = 0.0038 Å) by 0.1877 (18) Å. The plane of the phenyl substituent is practically perpendicular to that of the planar part of the five-membered ring, with a dihedral angle of 87.01 (5)°. In the crystal, mol­ecules are linked by weak C—H⋯O hydrogen bonds, forming inversion dimers. The dimers are linked by further C—H⋯O hydrogen bonds, as well as carbon­yl–carbonyl attractive inter­actions [O⋯C = 3.2879 (19) Å], forming a three-dimensional framework structure.

## Related literature   

For general background to the properties of α-substituted cyclic imides, see: Chen *et al.* (1951 ▶, 2014 ▶); Vida & Gerry (1977 ▶); Kuhnert-Brandstätter & Bösch (1978 ▶); Sigler *et al.* (2001 ▶); Lin *et al.* (2012 ▶). For the crystal structures of some succinimide derivatives, see: Argay & Carstensen-Oeser (1973 ▶); Argay & Kálmán (1973 ▶); Argay & Seres (1973 ▶); Kwiatkowski & Karolak-Wojciechowska (1992 ▶); Khrustalev *et al.* (2014 ▶). For carbon­yl–carbonyl inter­actions, see: Allen *et al.* (1998 ▶).
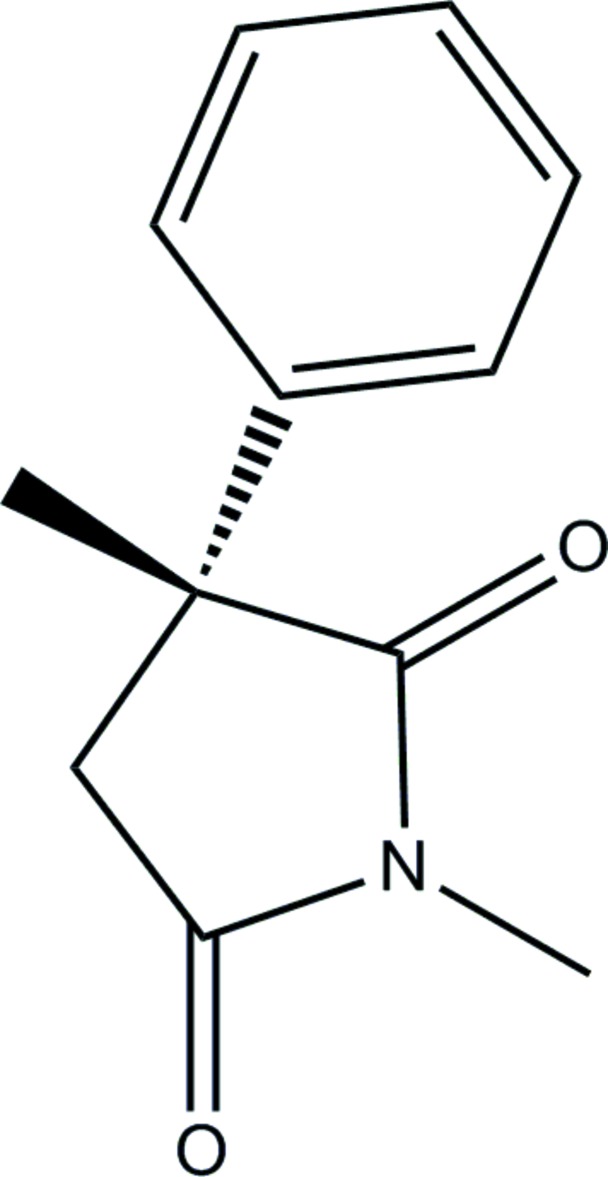


## Experimental   

### Crystal data  

C_12_H_13_NO_2_
*M* *_r_* = 203.23
Monoclinic, 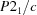

*a* = 10.517 (5) Å
*b* = 7.383 (3) Å
*c* = 13.568 (6) Å
β = 102.332 (6)°
*V* = 1029.2 (8) Å^3^
*Z* = 4
Mo *K*α radiation
μ = 0.09 mm^−1^
*T* = 100 K
0.45 × 0.35 × 0.25 mm

### Data collection  

Bruker SMART APEXII CCD area-detector diffractometer
Absorption correction: multi-scan (*SADABS*; Bruker, 2005 ▶) *T*_min_ = 0.961, *T*_max_ = 0.978
11948 measured reflections
3170 independent reflections
2401 reflections with *I* > 2σ(*I*)
*R*_int_ = 0.039

### Refinement  

*R*[*F* ^2^ > 2σ(*F* ^2^)] = 0.043
*wR*(*F* ^2^) = 0.117
*S* = 1.04
3170 reflections
138 parameters
H-atom parameters constrained
Δρ_max_ = 0.31 e Å^−3^
Δρ_min_ = −0.20 e Å^−3^

### 

Data collection: *APEX2* (Bruker, 2005 ▶); cell refinement: *SAINT* (Bruker, 2005 ▶); data reduction: *SAINT*; program(s) used to solve structure: *SHELXS97* (Sheldrick, 2008 ▶); program(s) used to refine structure: *SHELXL97* (Sheldrick, 2008 ▶); molecular graphics: *SHELXTL* (Sheldrick, 2008 ▶); software used to prepare material for publication: *SHELXTL*.

## Supplementary Material

Crystal structure: contains datablock(s) global, I. DOI: 10.1107/S1600536814016717/su2755sup1.cif

Structure factors: contains datablock(s) I. DOI: 10.1107/S1600536814016717/su2755Isup2.hkl

Supporting information file. DOI: 10.1107/S1600536814016717/su2755Isup3.cml

**Fig. 1.**
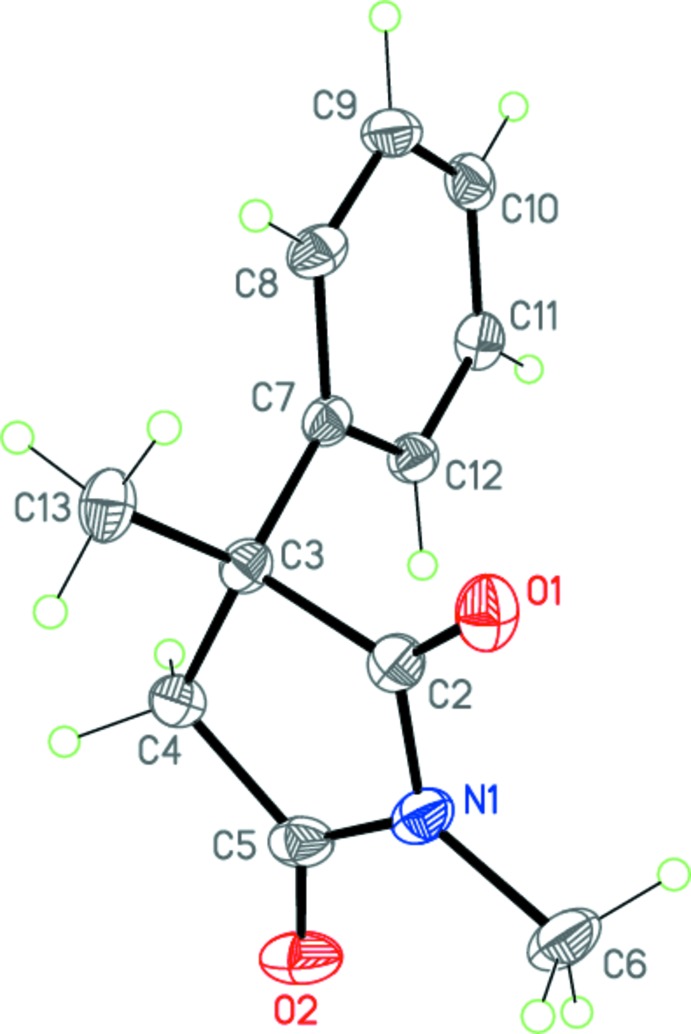
Molecular structure of the title molecule, with atom labelling. Displacement ellipsoids are drawn at the 50% probability level.

. DOI: 10.1107/S1600536814016717/su2755fig1.tif
Mol­ecular structure of the title mol­ecule, with atom labelling. Displacement ellipsoids are drawn at the 50% probability level.

**Fig. 2.**
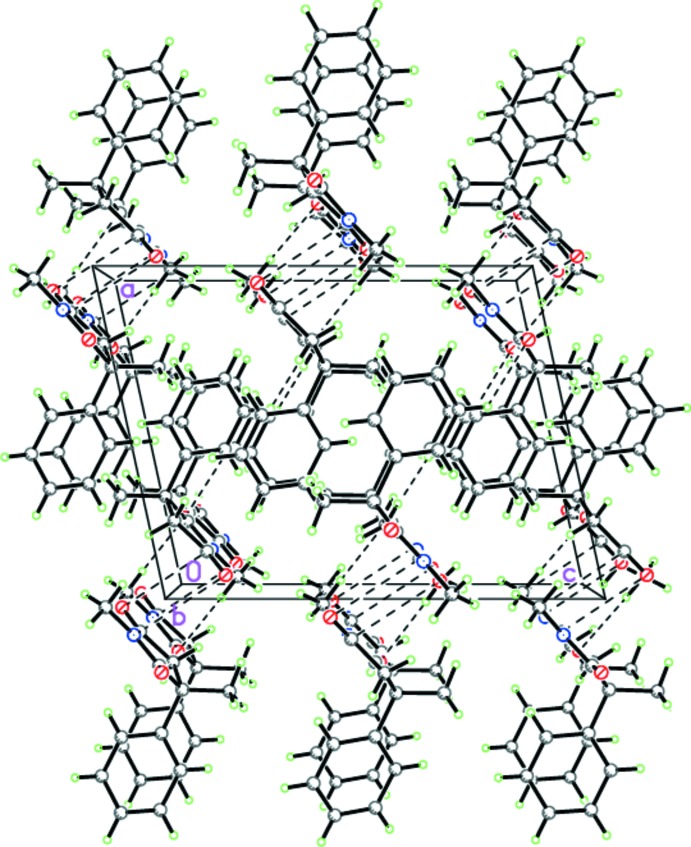
A view along the b axis of the crystal packing of the title compound. The C–H···O hydrogen bonds (see Table 1 for details) and attractive C═ O···C═O interactions are shown as dashed lines.

. DOI: 10.1107/S1600536814016717/su2755fig2.tif
A view along the b axis of the crystal packing of the title compound. The C–H⋯O hydrogen bonds (see Table 1 for details) and attractive C=O⋯C=O inter­actions are shown as dashed lines.

CCDC reference: 1015037

Additional supporting information:  crystallographic information; 3D view; checkCIF report

## Figures and Tables

**Table 1:** Hydrogen-bond geometry (Å, °)

*D*—H⋯*A*	*D*—H	H⋯*A*	*D*⋯*A*	*D*—H⋯*A*
C6—H6*C*⋯O1^i^	0.98	2.55	3.5264 (19)	178
C11—H11⋯O1^ii^	0.95	2.57	3.2744 (17)	132

Symmetry codes: (i) 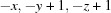; (ii) 
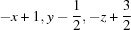
.

## supplementary crystallographic information

### S1. Comment

The potent antiepileptic properties of α-substituted cyclic imides have been known for over fifty years (Chen *et al.*, 1951; Vida & Gerry, 1977). For instance, 1,3-dimethyl-3-phenylpyrrolidine-2,5-dione (methsuximide) is a broad-spectrum anticonvulsant, valuable in the treatment of medically intractable epilepsy (Sigler *et al.*, 2001). *In vivo*, methsuximide is rapidly converted into its active metabolite, 3-methyl-3-phenylpyrrolidine-2,5-dione (α-methyl-α-phenylsuccinimide). Very recently we have studied the solid-state properties and crystal structures of racemic and homochiral forms of α-methyl-α-phenylsuccinimide (Khrustalev *et al.*, 2014; Chen *et al.*, 2014). Moreover, we have found and described the different polymorphic modifications of this compound (Khrustalev *et al.*, 2014). In this paper we report crystal structure of 1,3-dimethyl-3-phenylpyrrolidine-2,5-dione (methsuximide, trade-mark is Celontin).

The molecule of the title compound, C_12_H_13_NO_2_, contains the five-membered ring in a flattened *envelope* conformation; the C3 carbon atom is out of the mean plane passed through the other atoms of the ring (r.m.s. deviation is 0.0038) by 0.1877 (18) Å (Fig. 1). A similar conformation of the five-membered ring was also observed in other *N*-substituted succinimide derivatives (Argay & Seres, 1973; Kwiatkowski & Karolak-Wojciechowska, 1992). It should be noted that the five-membered ring in the *N*-unsubstituted succinimide derivatives adopts almost planar conformation (Argay & Kálmán, 1973; Argay & Carstensen-Oeser, 1973; Khrustalev *et al.*, 2014). The phenyl substituent is practically perpendicular to the five-membered ring; the dihedral angle between the planar part of the five-membered ring and the phenyl plane is 87.01 (5) °.

In the crystal, the molecules are linked by weak intermolecular C–H···O hydrogen bonds (Table 1) as well as carbonyl-carbonyl C5═ O2···C5^i^═O2^i^ [C5···O2^i^ and O2···C5^i^ distances are 3.2879 (19) Å; symmetry code: (i) - *x*, - *y*, - *z+1*] attractive interactions (Allen *et al.*, 1998) into a three-dimensional framework (Fig. 2).

It is important to point out that atom O2 does not form any intermolecular C–H···O hydrogen bonds due to the above-mentioned carbonyl-carbonyl interactions. Interestingly, molecular docking indicates that methsuximide seems to be incapable of forming hydrogen bonds with its protein target(s), which may explain why methsuximide, unlike 3-methyl-3-phenylpyrrolidine-2,5-dione, does not inhibit the nicotinic acetylcholine receptor (Chen *et al.*, 2014).

Previously, the different polymorphic modifications for close analogs of the ethosuximide (Lin *et al.*, 2012) and phensuximide (Kuhnert-Brandstätter & Bösch, 1978) derivatives were found by powder X-ray diffraction, IR spectroscopy and DSC methods. In search of polymorphic modifications for methsuximide we have carried out the DSC study of the title compound. However, no peaks that correspond to phase transitions, except for melting, were observed.

### S2. Experimental

1,3-Dimethyl-3-phenylpyrrolidine-2,5-dione (methsuximide, reference standard grade) was obtained under a Material Transfer Agreement with Pfizer Inc. and used without any further purification. The single crystals of the title compound were grown by slow evaporation of an EtOH/H_2_O (3:1) solvent mixture at room temperature; m.p. = 328-332 K.

### S3. Refinement

All H atoms were placed in calculated positions, with C–H = 0.95 Å (phenyl-H), 0.98 Å (methyl-H) and 0.99 Å (methylene-H) and refined as riding atoms with *U*_iso_(H) = 1.5*U*_eq_(C-methyl) and = 1.2*U*_eq_(C) for other H atoms.

### Crystal data

**Table:**

C_12_H_13_NO_2_	*F*(000) = 432
*M**_r_* = 203.23	*D*_x_ = 1.312 Mg m^−^^3^
Monoclinic, *P*2_1_/*c*	Mo *K*α radiation, λ = 0.71073 Å
Hall symbol: -P 2ybc	Cell parameters from 2487 reflections
*a* = 10.517 (5) Å	θ = 4.3–30.4°
*b* = 7.383 (3) Å	µ = 0.09 mm^−^^1^
*c* = 13.568 (6) Å	*T* = 100 K
β = 102.332 (6)°	Prism, colourless
*V* = 1029.2 (8) Å^3^	0.45 × 0.35 × 0.25 mm
*Z* = 4	

### Data collection

**Table:**

Bruker SMART APEXII CCD area-detector diffractometer	3170 independent reflections
Radiation source: fine-focus sealed tube	2401 reflections with *I* > 2σ(*I*)
Graphite monochromator	*R*_int_ = 0.039
φ and ω scans	θ_max_ = 30.7°, θ_min_ = 4.3°
Absorption correction: multi-scan (*SADABS*; Bruker, 2005)	*h* = −14→15
*T*_min_ = 0.961, *T*_max_ = 0.978	*k* = −10→10
11948 measured reflections	*l* = −19→19

### Refinement

**Table:**

Refinement on *F*^2^	Primary atom site location: structure-invariant direct methods
Least-squares matrix: full	Secondary atom site location: difference Fourier map
*R*[*F*^2^ > 2σ(*F*^2^)] = 0.043	Hydrogen site location: inferred from neighbouring sites
*wR*(*F*^2^) = 0.117	H-atom parameters constrained
*S* = 1.04	*w* = 1/[σ^2^(*F*_o_^2^) + (0.051*P*)^2^ + 0.2557*P*] where *P* = (*F*_o_^2^ + 2*F*_c_^2^)/3
3170 reflections	(Δ/σ)_max_ < 0.001
138 parameters	Δρ_max_ = 0.31 e Å^−^^3^
0 restraints	Δρ_min_ = −0.20 e Å^−^^3^

### Special details

**Table:**

Geometry. All esds (except the esd in the dihedral angle between two l.s. planes) are estimated using the full covariance matrix. The cell esds are taken into account individually in the estimation of esds in distances, angles and torsion angles; correlations between esds in cell parameters are only used when they are defined by crystal symmetry. An approximate (isotropic) treatment of cell esds is used for estimating esds involving l.s. planes.
Refinement. Refinement of F^2^ against ALL reflections. The weighted R-factor wR and goodness of fit S are based on F^2^, conventional R-factors R are based on F, with F set to zero for negative F^2^. The threshold expression of F^2^ > 2sigma(F^2^) is used only for calculating R-factors(gt) etc. and is not relevant to the choice of reflections for refinement. R-factors based on F^2^ are statistically about twice as large as those based on F, and R- factors based on ALL data will be even larger.

### Fractional atomic coordinates and isotropic or equivalent isotropic displacement parameters (Å^2^)

**Table:**

	*x*	*y*	*z*	*U*_iso_*/*U*_eq_	
O1	0.22108 (8)	0.49025 (12)	0.54547 (6)	0.0254 (2)	
O2	0.05261 (9)	−0.03777 (14)	0.63342 (7)	0.0329 (2)	
N1	0.12281 (9)	0.24733 (15)	0.60341 (7)	0.0230 (2)	
C2	0.21177 (11)	0.32854 (16)	0.55604 (8)	0.0197 (2)	
C3	0.29566 (11)	0.18081 (15)	0.52209 (8)	0.0178 (2)	
C4	0.22286 (11)	0.00564 (16)	0.53825 (9)	0.0213 (2)	
H4A	0.1797	−0.0476	0.4726	0.026*	
H4B	0.2840	−0.0846	0.5761	0.026*	
C5	0.12347 (11)	0.06034 (18)	0.59769 (8)	0.0232 (2)	
C6	0.03228 (12)	0.3509 (2)	0.64901 (10)	0.0320 (3)	
H6A	0.0782	0.4530	0.6870	0.048*	
H6B	−0.0035	0.2726	0.6948	0.048*	
H6C	−0.0387	0.3967	0.5960	0.048*	
C7	0.43011 (10)	0.19242 (14)	0.59288 (8)	0.0165 (2)	
C8	0.53672 (11)	0.26871 (15)	0.56295 (9)	0.0207 (2)	
H8	0.5273	0.3141	0.4963	0.025*	
C9	0.65706 (11)	0.27933 (16)	0.62961 (10)	0.0236 (3)	
H9	0.7288	0.3323	0.6081	0.028*	
C10	0.67313 (12)	0.21356 (16)	0.72690 (10)	0.0233 (2)	
H10	0.7558	0.2194	0.7718	0.028*	
C11	0.56728 (11)	0.13896 (16)	0.75822 (9)	0.0214 (2)	
H11	0.5772	0.0940	0.8250	0.026*	
C12	0.44715 (11)	0.12992 (15)	0.69216 (8)	0.0186 (2)	
H12	0.3750	0.0803	0.7147	0.022*	
C13	0.29842 (13)	0.21253 (17)	0.41107 (9)	0.0240 (3)	
H13A	0.3302	0.3352	0.4027	0.036*	
H13B	0.2104	0.1988	0.3697	0.036*	
H13C	0.3564	0.1239	0.3897	0.036*	

### Atomic displacement parameters (Å^2^)

**Table:**

	*U*^11^	*U*^22^	*U*^33^	*U*^12^	*U*^13^	*U*^23^
O1	0.0279 (5)	0.0234 (4)	0.0231 (4)	0.0064 (3)	0.0012 (3)	−0.0014 (3)
O2	0.0212 (4)	0.0458 (6)	0.0329 (5)	−0.0032 (4)	0.0084 (4)	0.0098 (4)
N1	0.0173 (5)	0.0324 (6)	0.0196 (5)	0.0046 (4)	0.0046 (4)	0.0000 (4)
C2	0.0172 (5)	0.0258 (6)	0.0147 (5)	0.0035 (4)	0.0002 (4)	0.0000 (4)
C3	0.0188 (5)	0.0192 (5)	0.0159 (5)	0.0011 (4)	0.0047 (4)	0.0008 (4)
C4	0.0189 (5)	0.0236 (6)	0.0214 (5)	−0.0023 (4)	0.0041 (4)	−0.0010 (4)
C5	0.0160 (5)	0.0340 (6)	0.0186 (5)	−0.0003 (4)	0.0016 (4)	0.0034 (5)
C6	0.0210 (6)	0.0474 (8)	0.0292 (6)	0.0099 (5)	0.0086 (5)	−0.0032 (6)
C7	0.0173 (5)	0.0153 (5)	0.0179 (5)	0.0017 (4)	0.0058 (4)	−0.0001 (4)
C8	0.0221 (6)	0.0193 (5)	0.0232 (5)	0.0014 (4)	0.0102 (4)	0.0031 (4)
C9	0.0186 (5)	0.0186 (5)	0.0358 (7)	−0.0012 (4)	0.0113 (5)	−0.0025 (5)
C10	0.0193 (5)	0.0196 (5)	0.0295 (6)	0.0026 (4)	0.0023 (4)	−0.0066 (4)
C11	0.0232 (6)	0.0215 (5)	0.0189 (5)	0.0034 (4)	0.0033 (4)	−0.0015 (4)
C12	0.0190 (5)	0.0183 (5)	0.0195 (5)	−0.0002 (4)	0.0061 (4)	0.0000 (4)
C13	0.0316 (6)	0.0249 (6)	0.0158 (5)	0.0015 (5)	0.0061 (5)	0.0000 (4)

### Geometric parameters (Å, º)

**Table:**

O1—C2	1.2089 (15)	C7—C8	1.3907 (16)
O2—C5	1.2122 (15)	C7—C12	1.3987 (16)
N1—C2	1.3802 (15)	C8—C9	1.3918 (18)
N1—C5	1.3828 (18)	C8—H8	0.9500
N1—C6	1.4580 (16)	C9—C10	1.3826 (19)
C2—C3	1.5342 (16)	C9—H9	0.9500
C3—C13	1.5308 (17)	C10—C11	1.3876 (17)
C3—C7	1.5328 (16)	C10—H10	0.9500
C3—C4	1.5422 (16)	C11—C12	1.3859 (16)
C4—C5	1.5055 (17)	C11—H11	0.9500
C4—H4A	0.9900	C12—H12	0.9500
C4—H4B	0.9900	C13—H13A	0.9800
C6—H6A	0.9800	C13—H13B	0.9800
C6—H6B	0.9800	C13—H13C	0.9800
C6—H6C	0.9800		
			
C2—N1—C5	113.30 (10)	H6B—C6—H6C	109.5
C2—N1—C6	122.61 (11)	C8—C7—C12	118.00 (10)
C5—N1—C6	123.98 (11)	C8—C7—C3	122.21 (10)
O1—C2—N1	124.36 (11)	C12—C7—C3	119.76 (10)
O1—C2—C3	126.88 (11)	C7—C8—C9	120.73 (11)
N1—C2—C3	108.76 (10)	C7—C8—H8	119.6
C13—C3—C7	113.53 (9)	C9—C8—H8	119.6
C13—C3—C2	108.64 (9)	C10—C9—C8	120.63 (11)
C7—C3—C2	106.55 (9)	C10—C9—H9	119.7
C13—C3—C4	112.49 (9)	C8—C9—H9	119.7
C7—C3—C4	112.17 (9)	C9—C10—C11	119.29 (11)
C2—C3—C4	102.63 (9)	C9—C10—H10	120.4
C5—C4—C3	105.90 (10)	C11—C10—H10	120.4
C5—C4—H4A	110.6	C12—C11—C10	120.09 (11)
C3—C4—H4A	110.6	C12—C11—H11	120.0
C5—C4—H4B	110.6	C10—C11—H11	120.0
C3—C4—H4B	110.6	C11—C12—C7	121.24 (10)
H4A—C4—H4B	108.7	C11—C12—H12	119.4
O2—C5—N1	124.28 (12)	C7—C12—H12	119.4
O2—C5—C4	127.63 (12)	C3—C13—H13A	109.5
N1—C5—C4	108.07 (10)	C3—C13—H13B	109.5
N1—C6—H6A	109.5	H13A—C13—H13B	109.5
N1—C6—H6B	109.5	C3—C13—H13C	109.5
H6A—C6—H6B	109.5	H13A—C13—H13C	109.5
N1—C6—H6C	109.5	H13B—C13—H13C	109.5
H6A—C6—H6C	109.5		
			
C5—N1—C2—O1	174.37 (10)	C3—C4—C5—O2	−173.64 (11)
C6—N1—C2—O1	−2.00 (17)	C3—C4—C5—N1	7.94 (12)
C5—N1—C2—C3	−6.64 (13)	C13—C3—C7—C8	15.33 (15)
C6—N1—C2—C3	176.99 (10)	C2—C3—C7—C8	−104.22 (12)
O1—C2—C3—C13	−50.84 (15)	C4—C3—C7—C8	144.23 (10)
N1—C2—C3—C13	130.20 (10)	C13—C3—C7—C12	−166.67 (10)
O1—C2—C3—C7	71.83 (14)	C2—C3—C7—C12	73.78 (12)
N1—C2—C3—C7	−107.13 (10)	C4—C3—C7—C12	−37.77 (13)
O1—C2—C3—C4	−170.14 (11)	C12—C7—C8—C9	1.08 (16)
N1—C2—C3—C4	10.91 (11)	C3—C7—C8—C9	179.11 (10)
C13—C3—C4—C5	−127.64 (10)	C7—C8—C9—C10	0.25 (17)
C7—C3—C4—C5	102.92 (10)	C8—C9—C10—C11	−0.98 (17)
C2—C3—C4—C5	−11.07 (11)	C9—C10—C11—C12	0.36 (17)
C2—N1—C5—O2	−179.41 (11)	C10—C11—C12—C7	1.00 (17)
C6—N1—C5—O2	−3.10 (18)	C8—C7—C12—C11	−1.70 (16)
C2—N1—C5—C4	−0.93 (13)	C3—C7—C12—C11	−179.79 (10)
C6—N1—C5—C4	175.39 (10)		

### Hydrogen-bond geometry (Å, º)

**Table:**

*D*—H···*A*	*D*—H	H···*A*	*D*···*A*	*D*—H···*A*
C6—H6*C*···O1^i^	0.98	2.55	3.5264 (19)	178
C11—H11···O1^ii^	0.95	2.57	3.2744 (17)	132

Symmetry codes: (i) −*x*, −*y*+1, −*z*+1; (ii) −*x*+1, *y*−1/2, −*z*+3/2.
