# Supplementary figures and images for: Crystal structure of 1,3-dimethyl-3-phenyl­pyrrolidine-2,5-dione: a clinically used anti­convulsant
Source: Acta Crystallogr Sect E Struct Rep Online. 2014 Aug 1;70(Pt 9):o942–3. doi: 10.1107/S1600536814016717 (PMC4186160; doi:10.1107/S1600536814016717)

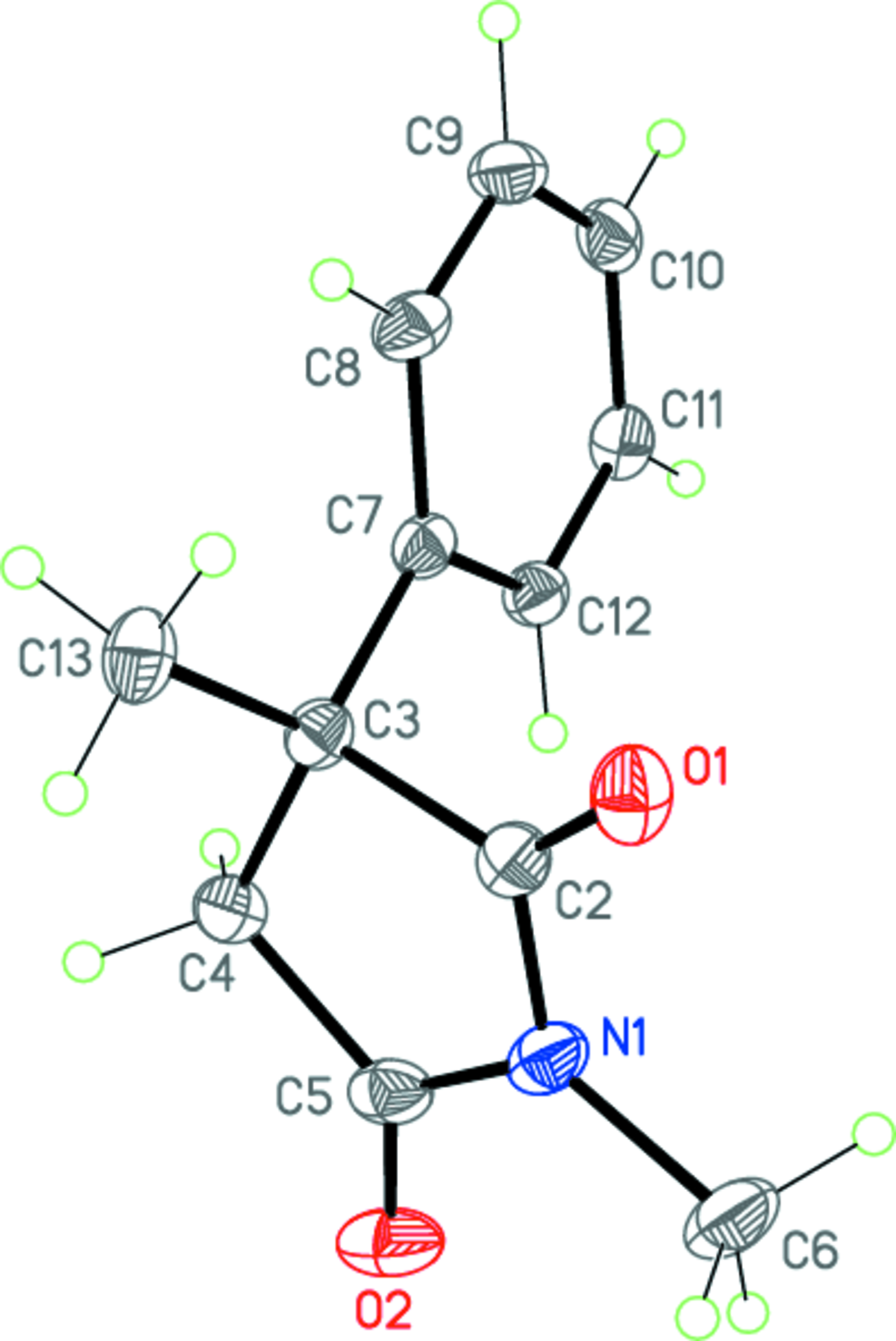

Supplement: Supplementary file 4 [file e-70-0o942-fig1.tif]

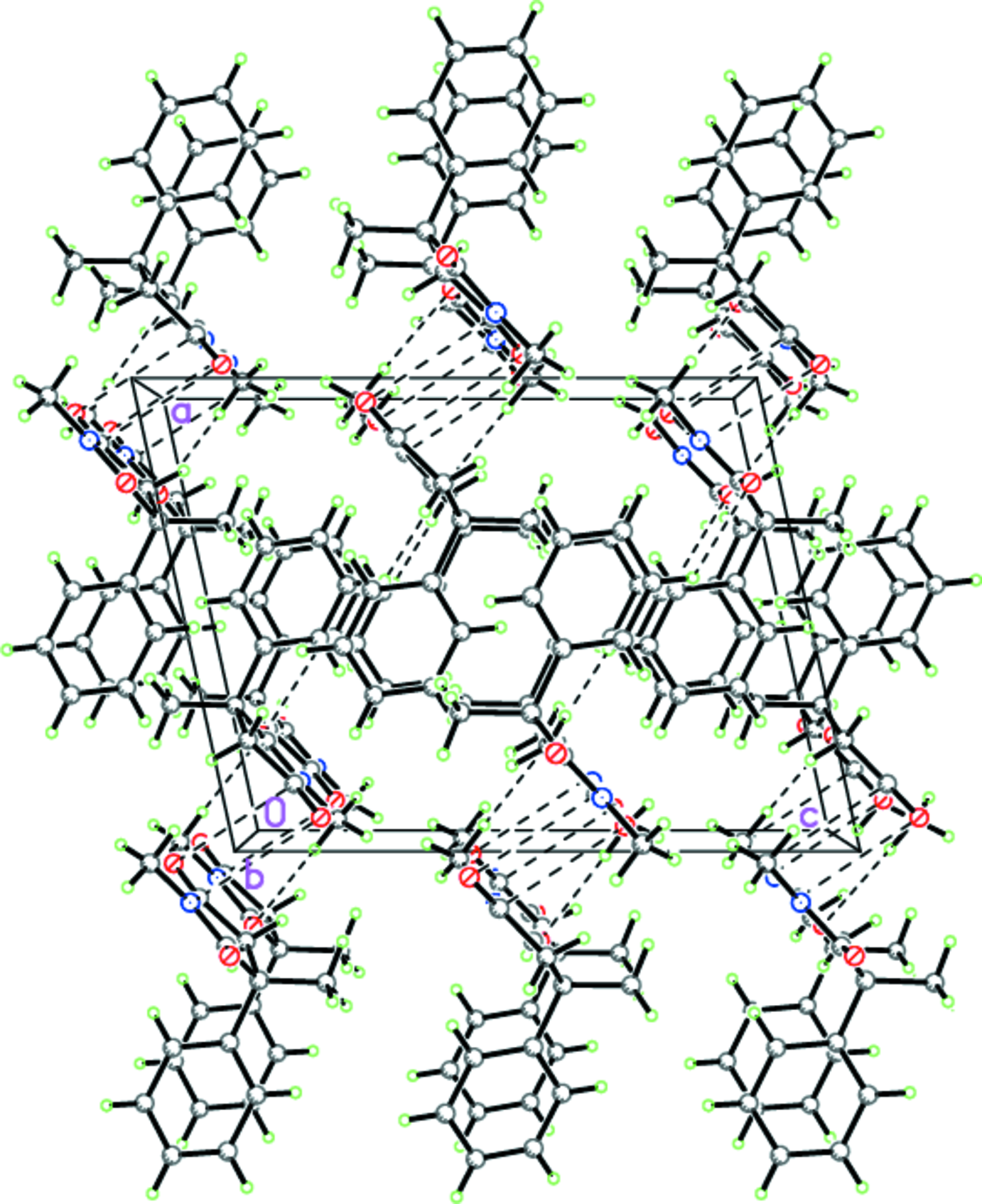

Supplement: Supplementary file 5 [file e-70-0o942-fig2.tif]
